# Advances in Fungal Peptide Vaccines

**DOI:** 10.3390/jof6030119

**Published:** 2020-07-25

**Authors:** Leandro B. R. Da Silva, Carlos P. Taborda, Joshua D. Nosanchuk

**Affiliations:** 1 Instituto de Ciencias Biomedicas, Departamento de Microbiologia, Universidade de Sao Paulo, Sao Paulo 05508-000, Brazil; leandrobr87@usp.br (L.B.R.D.S.); taborda@usp.br (C.P.T.); 2 Departments of Medicine (Division of Infectious Diseases) and Microbiology and Immunology, Albert Einstein College of Medicine, New York, NY 10461, USA; 3 Instituto de Medicina Tropical de Sao Paulo, Laboratorio de Micologia Medica, Departamento de Dermatologia, Faculdade de Medicina, Universidade de Sao Paulo, Sao Paulo 05403-000, Brazil

**Keywords:** fungal, vaccine, peptides, *paracoccidioides*, *sporothrix*, *aspergillus*, *candida*, *coccidioides*, dendritic cells

## Abstract

Vaccination is one of the greatest public health achievements in the past century, protecting and improving the quality of life of the population worldwide. However, a safe and effective vaccine for therapeutic or prophylactic treatment of fungal infections is not yet available. The lack of a vaccine for fungi is a problem of increasing importance as the incidence of diverse species, including *Paracoccidioides, Aspergillus, Candida, Sporothrix*, and *Coccidioides*, has increased in recent decades and new drug-resistant pathogenic fungi are emerging. In fact, our antifungal armamentarium too frequently fails to effectively control or cure mycoses, leading to high rates of mortality and morbidity. With this in mind, many groups are working towards identifying effective and safe vaccines for fungal pathogens, with a particular focus of generating vaccines that will work in individuals with compromised immunity who bear the major burden of infections from these microbes. In this review, we detail advances in the development of vaccines for pathogenic fungi, and highlight new methodologies using immunoproteomic techniques and bioinformatic tools that have led to new vaccine formulations, like peptide-based vaccines.

## 1. Introduction

Although preceded by variolation in China as early as the 15th century, the development of standardized vaccines began in the 18th century [1]. From the beginning, vaccine development has been focused on using whole attenuated or inactivated microorganisms or fractions of microbes [1]. Remarkably, these methodologies are still routinely used in modern vaccinology. More recently, with advances in technology and a more detailed understanding of immunology, newer innovative methods are being applied for vaccine development. Our current vaccine armamentarium includes vaccines against diverse lethal viral and bacterial diseases, but there is no vaccine against a fungal disease. The absence of mass market appeal has been presented as the major obstacle in fungal vaccine development [2,3,4,5,6]. However, this situation is changing rapidly as the incidence of invasive mycoses has increased with the rising numbers of individuals with increased risk for fungal disease, including cancer patients receiving chemotherapy, bone marrow transplant recipients, individuals with acquired immune deficiency syndrome (AIDS), individuals treated with immune function inhibitors, and others patients with different types of immunosuppression [7,8,9]. Additionally, certain fungi, such as *Candida auris*, an emerging multi-drug resistant pathogen, have received significant global media attention. Importantly, systemic mycoses are among the leading causes of death and morbidity in the USA, totaling more than 1.6 million deaths [10,11], with annual costs over $7.2 billion dollars [12,13,14]. Hence, the increased incidence of mycoses, the emergence of multi-drug resistant species, and the rising costs associated with combatting these diseases have recently led to significantly greater visibility of fungal diseases worldwide.

The global burden of invasive mycoses is massive. For example, there are at least ~250,000 cases of invasive aspergillosis, 3,000,000 cases of chronic pulmonary aspergillosis, ~223,100 cases of cryptococcal meningitis, ~700,000 cases of invasive candidiasis, ~500,000 cases of *Pneumocystis jirovecii* pneumonia, ~100,000 cases of disseminated histoplasmosis, ~10,000,000 cases of fungal asthma, and ~1,000,000 cases of fungal keratitis annually [15,16,17]. Why, with the increase in the number of cases and the immense medical need, have more than two centuries of vaccine research failed to produce a single therapeutic or prophylactic vaccine for a mycosis? Vaccines, in addition to preventing lethal diseases, improve people’s quality of life [18,19], which is particularly notable for fungal diseases that frequently require protracted durations of antifungal drugs that have diverse toxicities and costs. Therefore, we will briefly discuss general issues in fungal vaccine development and then we will focus on some novel and conceptual advances in the field of peptide vaccines against fungal infections, which may simplify and accelerate the achievement of a safe and effective antifungal vaccine that is effective in both immunocompetent and immunologically suppressed individuals.

## 2. Fungal Vaccine: Some Challenges

One of the first and most difficult challenges in working with fungal vaccines is the need to determine the target population in which a vaccine is applied, as many invasive mycoses have a predilection for causing disease in immunosuppressed individuals [7,8,9]. Specific knowledge is required regarding the type of protective response necessary to combat a specific fungus and then there is a need to translate this information into a formulation that remains safe and effective in an immunocompromised host [20,21].

Another obstacle on the pathway to developing vaccines against fungal infection is the complexity of the fungal cell. Fungi are eukaryotic, and pathogenic species have marked differences and similarities with human cells. Fungal cells have a double layer of protection: an inner plasma membrane and an outer cell wall [22]. The plasma membrane is a phospholipid bilayer that may vary in composition, due to the presence of specific fungal sterols in different species. Ergosterol, which is similar to human cholesterol, is particularly important for membrane fluidity and it is essential for viability [23]. The cell wall is generally organized as a scaffold of carbohydrate polymers to which a variety of proteins and other components are added, creating a strong but elastic structure [22]. Although there are diverse variations in polysaccharide composition across species, there are conserved components, such as a core of branched β-1,3-glucan-chitin [24]. Thus, the fact that fungi have preserved compounds in both the cell wall and plasma membrane makes it theoretically possible to develop a universal vaccine, where the presence of a common antigen among closely-related and/or disparate pathogens could be used to protect against different mycosis or even disease caused by others microorganisms [25,26]. For example, a β-glucan laminarin has demonstrated protection against infection by *Candida* and *Aspergillus* species by means of growth-inhibiting antibodies, particularly when conjugated with the diphtheria toxoid CRM197 carrier protein [27,28,29].

An interesting and intriguing aspect of fungal vaccines is the apparent existence of two major immunological mechanisms for achieving protection. The immune responses that have received the most study for fungal infection are a Th1 and/or Th17-based response and antibody-mediated immunity. Although both immune processes cooperate for the final protective outcome, the mechanisms are different. In particular, Th1 and/or Th17 immune response mediate protection indirectly, promoting an inflammatory response with recruitment of soluble (antimicrobial peptides, cytokines, chemokines) and cellular (macrophages, neutrophils) effectors that are responsible for the elimination or control of the fungal cells at the site of infection [30]. In contrast, antibodies can mediate protection not only by classical opsonization and complement activation, but also by direct neutralization of factors such as adhesins or enzymes, which are a critical step for infection, fungal growth, inhibiting fungal escape from host immunity, or even directly killing the fungus [31,32,33]. Additionally, antibody binding to the fungal cell surface can directly regulate biological processes in the bound cells [34,35,36]. Recently, Boniche et al., (2020) described the approaches in immunotherapy against systemic mycosis using antibodies and the importance of this method for prospectively protecting immunocompromised host with defective cellular effectors.

Vaccine protection starts with the injection of an antigen source (live, inactivated, subunits, nucleic acids) that will be taken up/recognized by professional antigen-presenting cells (APCs) (macrophages or dendritic cells). After antigen phagocytosis, the APCs migrate to lymphoid organs where they interact with and present the antigen to lymphocytes. These lymphocytes are activated upon recognizing the antigen and by concomitant receipt of appropriate co-stimulatory signals, and the activated lymphocytes then produce a cell specific immune response. Activated B cells produce antibodies (IgG, IgM, IgA, IgE) that can target the invading fungal cells or otherwise modify immune cell responses. T-Cells are the major representative of cell-mediated immunity; activated Cytotoxic T-lymphocytes (CTLs, also called cytotoxic T-cells) can directly kill fungi, and T-helper cells (Th1-type) activate macrophages to enhance their capacity to kill intracellular pathogens. Furthermore, some of the B- and T-cells maintain themselves for many years as memory B- and T-cells such that they can rapidly activate and clonally expand when they encounter specific fungal antigens in the future and effectively combat the invading fungus [37,38,39]. A vaccine can be comprised by a live attenuated or inactivated microorganism or by one or more antigens. Antigens may be derived from the microbe, such as nucleic acids, proteins, carbohydrates or polysaccharides, and their efficacy can be enhanced by using targeted components of these structures, which is the case with peptide vaccines.

## 3. Fungal Peptide Vaccine

As discussed, immunization or vaccination using live-attenuated or inactivated pathogens (virus, bacteria, fungal, etc.) have been used for the induction of antigen-specific responses to protection against subsequent experimental infections. However, whole microbes contain thousands of distinct antigens and many are unnecessary for the induction of protective immune responses; moreover, some may induce unwanted responses, such as allergenic and/or reactogenic responses. These concerns have led to studies of subunits, such as a protein, from pathogens as vaccine candidates [40,41]. However, proteins are also relatively large and display many antigenic epitopes, which can also lead to adverse activities along with the induction of protective immune response. Therefore, peptide vaccines have been explored for their ability to induce desirable T cell and B cell-mediated immune response to highly defined, specific epitopes [42].

The first indication that a peptide vaccine could modify host-pathogen interactions arose from studies on tobacco mosaic virus in 1963, when Anderer demonstrated that conjugation of a hexapeptide derived from viral coat protein and coupled with bovine serum albumin could induce neutralizing antibodies to the intact virus [43]. Subsequently, Anderer demonstrated that synthetic tri-, tetra-, penta, or hexapetides also effectively generated neutralizing antibodies [44]. However, the work by Langebeheim and colleagues (1976) was the first to demonstrate that synthetic peptides derived from the coat protein of bacteriophage MS2 could induce antibodies that were as effective as those generated against the intact protein for neutralizing the bacteriophage. These discoveries together with ongoing technological advances, particularly in refining techniques for sequencing proteins and synthesizing peptides [45], spurred a marked increase in experimental peptide vaccine research in the 1980s [46]. Studies to date have demonstrated that engineered peptide vaccines can generally be considered as safe and cost effective when compared to conventional vaccines. However, the peptide’s small size means that they are weakly immunogenic, such that they require transport molecules, which have the dual roles of serving as an adjuvant and promoting chemical stability [47].

Currently, the PubMed database (NCBI) contains thousands of reports on clinical studies of peptide vaccines for therapeutic or prophylactic use for diseases such as HIV, hepatitis C virus (HCV), hepatitis B virus (HBV), cytomegalovirus (CMV), influenza, tuberculosis, malaria, pneumonia, genital herpes, and cancer, among others. However, it is also possible to find hundreds of studies of peptide vaccines for the treatment of fungal infection such as coccidioidomycosis, histoplasmosis, sporotrichosis, paracoccidioidomycosis, blastomycosis, aspergillosis, cryptococcosis, candidiasis, and other mycoses. The focus in this review is on new studies on engineering-based peptides for the treatment of fungal infections that are particularly due to *Paracoccidioides, Aspergillus, Candida, Sporothrix*, and *Coccidioides*. Table 1 provides an overview of the data from the major references detailed in this review.

### 3.1. Paracoccidioidomycosis (PCM)

Remarkably, fungal peptide vaccines are most advanced for the treatment of paracoccidioidomycosis, which is a neglected fungal disease restricted to Latin America [48]. Among the most promising treatments for Paracoccidioidomycosis is the vaccine using peptide 10 (P 10). This peptide was mapped based on the sequence of gp43, the main diagnostic antigen of *P. brasiliensis*. P10 is responsible for inducing lymphoproliferation and contains a major CD4^+^ specific T cell epitope and elicits an IFN-γ-dependent Th1 immune response, which is considered a protective and effective immune response against the infection with fungi of the genus *Paracoccidioides* [49,50,51,52]. Immunization with P10 proved to be protective in prophylactic and therapeutic murine infection models when injected with complete Freud’s adjuvant [49]. Significantly, the peptide also protected against lethal infection in a model using immunosuppressed mice [53]. Rittner and collaborators (2012), using a gene therapy approach with a pcDNA3 expression vector encoding P10, demonstrated that this therapeutic DNA vaccine, given prior to or after infection, significantly reduced pulmonary fungal burdens in a murine infection model [54]. Also in 2012, Magalhães and collaborators showed that adoptive transfer of dendritic cells (DCs) pulsed with P10, either prior to or after infections, significantly protected mice from *P. brasiliensis* [55]. Building on these DC results, studies were also performed on immunocompromised animals. Bone marrow-derived dendritic cells (BMDCs) pulsed with P10 efficiently reduced the pulmonary fungal burdens of immunosuppressed mice previously infected with *P. brasiliensis* and also preserved lung tissue by decreasing cellular infiltration into the organ [56]. Further work demonstrated that P10 was able to activate and modulate both BMDCs and monocyte-derived dendritic cells (MoDCs), and MoDCs pulsed with P10 similarly protected against pulmonary infection by *P. brasiliensis*, which is promising as this treatment most closely mirrors what would be administered to a patient with paracoccidioidomycosis [57].

The majority of research on antibodies in paracoccidioidomycosis have focused on their use for serological diagnosis, particularly as high titers of antibodies that are generated by patients with the acute-subacute form of the disease, which is the more aggressive form [101]. However, studies on antibody-mediated immune system modulations in response to an experimental model of cryptococcus infection [102,103,104,105,106] have led to an interest in the generation of protective antibodies for mitigating infections with *Paracoccidioides*. In fact, there are polyclonal [107] and monoclonal [108] antibodies that are protective in paracoccidioidomycosis. However, no vaccine specific for generating a humoral response is established for the treatment of paracoccidioidomycosis. Notably, a recent analysis of extracellular antigens from *Paracoccidioides* species using immunoproteomic approaches combined with immunoprecipitation using B-cells followed by antigen identification by nanoUPLC-MS^E^-based proteomics demonstrated a variety of *Paracoccidioides* B-cell epitopes, common or specific to members in the species complex. Using bioinformatic tools, the proteins and the sequence of these epitopes from extracellular antigens were identified; however, these epitopes have not yet been tested. Nevertheless, this work highlights an opportunity for a new approach using synthetic peptides with the potential to stimulate antibody-mediated immune immunity [58].

### 3.2. Aspergillosis

Among the most feared fungal pathogens that are frequently clinically encountered are *Aspergillus* species, which are responsible for causing invasive aspergillosis as well as chronic bronchopulmonary aspergillosis. *Aspergillus fumigatus* is one of the most common species that notoriously causes infection in immunocompromised hosts, particularly in patients undergoing antineoplastic chemotherapy and those with organ transplants [109,110,111,112,113,114]. Although several laboratories have investigated the development of a safe and effective vaccine against aspergillosis and some promising results have been obtained in an experimental model using homologous proteins, crude extracts or recombinant allergens from Aspergillus [115,116,117,118], there is no vaccine against aspergillosis.

In silico assays, such as the use of artificial neural networks and immune epitope databases, facilitate the prediction of B cell epitopes and T cell MHC epitopes [119,120,121]. Subjecting *A. fumigatus* allergens to such analyses resulted in the identification of five potential allergic proteins (Asp f1, Asp f2, Asp f5, Asp f17, and Asp f34) with common B and T cell epitopes for both mice and humans [59]. Hence, these five proteins with high affinity binding to MHC class I or II epitopes could be used to characterize constituent peptides and develop vaccine candidates for invasive *Aspergillus* infections or therapeutics for allergy immunotherapy for chronic allergic bronchopulmonary aspergillosis. The promise of this approach is supported by prior work demonstrating that peptides from the protein Asp f1 stimulate the production of Th1 cytokines [60]. However, these proteins have not yet been validated as effective vaccine components.

### 3.3. Candidiasis

Candida species are the most frequently isolated fungal species in blood cultures worldwide and these opportunistic pathogens cause a wide range of infections. Disseminated bloodstream infection has an estimated mortality rate of 40–60% even with the use of antifungal drugs [122,123,124,125]. Additionally, *Candida* commonly cause vaginitis, oral thrush, and infections of the skin and nails. Despite the incredibly high overall disease incidence as well as the frequency and severity of invasive infections, there is no vaccine for *Candida* species. However, peptide-based vaccine strategies have been considered for over two decades for both prevention and protection thought active and passive immunization [2,126,127].

Several studies demonstrated that antibodies specific for the peptide Fba or peptide Met6, which were respectively derived from *C. albicans* cell surface fructose bisphosphate aldolase (Fab) or β-1,2–mannotriose [β-(Man)3] protein, were induced by a protective glycopeptide vaccine [33,61,62,63]. More recently, active immunization using DCs pulsed with either Fba peptide (YGKDVKDLFDYAQE) or Met6 peptide (PRIGGQRELKKITE) were protective in both neutropenic and immunocompetent mice [64]. Subsequently, a study of a synthetic 14-mer Fba peptide conjugated to each of the five peptides mimotopes from Met6 (PS2, PS31, PS28, PS55, and PS76) were tested to explore their protective capacity [65]. All five peptides mimotopes induced specific antibody responses, and immunization with three of the peptide conjugate vaccines protected against disseminated candidiasis in mice [65].

Another recent peptide vaccine-based approach was achieved using computational tools to identify immunologically active compounds to combat candidiasis. A screen of 6030 proteins identified in the proteome of *Candida albicans* (sc5314) [66] was undertaken to identify immunodominant HLA class I, HLA class II and linear and discontinuous B-cell epitopes. The screen identified 214 epitopes that were subjected to conservation analysis using 22 *C. albicans* strains with published sequenced genomes, and 18 peptides displayed 100% conservancy. The 18 peptides were then used to construct a multivalent recombinant protein to which they added a synthetic adjuvant called RS09. However, the investigators do not yet describe the efficacy of this polymeric vaccine, and it is notable that this type of protein peptide-base vaccine will only generate responses in patients with specific HLA haplotypes that are able to bind these particular peptide epitopes [67]. Nevertheless, this type of vaccine approach may generate effective immune response using well-defined minimal quantities of antigen, which may minimize unwanted side effects.

There is a growing literature on the production and release of fungal extracellular vesicles (EV), which occurs in both ascomycetes and basidiomycetes [68,69]. These EV contain large quantities of biologically functional compounds that are associated with virulence, including in EV from *Candida* [70]. Recent data highlights how these relatively stable EV can be used as safe source for diverse antigens, including peptides, for vaccine development as administration of *Candida* EV are protective in a murine systemic candidiasis infection model [71].

The vaccine proposal in the most advanced phase of study for combatting a fungal infection is NDV-3A [72]. This vaccine is based on *C. albicans* Als3p, which is a glycoprotein with an agglutinin-like sequence that is associated with virulence through effects on fungal adherence, invasion and biofilm formation [73,74]. The first version of this vaccine, NDV-3, was a His-tagged recombinant Als3 protein N-terminus (rAls3p-N), combined with alum, which was protective in a disseminated candidiasis experimental model [75,76,77,78]. In a Phase I clinical trial, NDV-3 was highly immunogenic and well-tolerated [78]. The more recent version of the vaccine, NDV-3A, was prepared with rAls3p-N without the His-tag, and again combined with alum [72]. An exploratory Phase 1b/2a study found that a single intramuscular dose of NDV-3 was safe and immunogenic. In a phase 2 randomized, double-blind, placebo-control trial, NDV-3A was administered to women with recurrent vulvovaginal candidiasis (RVVC) and the vaccine was found to be safe and vaccinated women rapidly developed both T and B cell responses to rAls3p-N. In what the authors describe as “unprecedented”, the vaccine effectively reduced the occurrence and frequency of vulvovaginal candidiasis (VVC) episodes for up to 12 months [72]. Additionally, serum from patients who responded to NDV-3A contained antibodies that prevented fungal adhesion and biofilm formation on plastic as well as fungal invasion of vaginal epithelial cells in vitro [79]. This group recently demonstrated that mice vaccinated with NDV-3A developed high titers of ant-rAls3-N antibodies and that the presence of these antibodies was sufficient to block *C. albicans* from colonizing jugular vein catheters [80]. The NDV-3A is a highly promising vaccine for treatment of RVVC as well as invasive candidiasis.

NDV-3A has also been tested against *C. auris*, which is an emerging, multi-drug resistant species [81,82] that has marked biological differences from *C. albicans* [83]. Homologs of the *C. albicans* Als3p, glycoprotein base of the NDV-3A vaccine, are present in isolates of different *C. auris* clades [84]. Preliminary studies have revealed that mice vaccinated with NDV-3A develop antibodies to Als3p and that these antibodies recognize *C. auris* yeast cells in vitro, block their ability to form biofilms, and improve macrophage-mediated fungal killing. In an in vivo murine model, NDV-3A effectively induced cross-reactive humoral and cellular immune responses that protected immunosuppressed mice who received a lethal challenge of *C. auris*. Furthermore, NDV-3A improved the efficacy of sub-therapeutic doses of micafungin [84]. Thus, the advanced development of NDV-3A is extremely promising for use not only against *C. albicans* but also against multidrug-resistant *C. auris*.

### 3.4. Sporotrichosis

The species that make up the Sporothrix complex (*Sporothrix schenckii*, *S. brasiliensis*, *S. globosa* and *S. luriei*) are distributed worldwide [128], Although cutaneous and lymphocutaneous forms are by far the most common disease manifestations, disseminated disease may occur in immunocompromised patients [129,130] and pulmonary disease may manifest after conidia or propagules of the fungus are inhaled [131]. Significantly, *S. brasiliensis* has an increased tendency to disseminate even in the absence of any immune defect [132]. Several countries have reported an increase in the number of cases of feline zoonotic transmission, and this epidemic is primarily due to *S. brasiliensis* in Brazil [133,134]. Although monoclonal antibodies have experimentally produced positive results [135,136], the search for new and more efficient treatment modalities is ongoing.

Given the increasing incidence and disease severity with *S. brasiliensis*, de Almeida and colleagues undertook a proteomic analysis using the in-silico prediction tools to identify peptides with high affinity to MHC class II. They identified seven peptides that met their criteria, which were synthesized and tested in mice. Three of the peptide vaccines induced proliferation of T cells sensitized by *S. brasilienis* in vitro. In subsequent in vivo experiments, immunization with each of the three peptides mixed in Freund’s incomplete adjuvant reduced the severity of subcutaneous sporotrichosis and one peptide induced the production of high levels of inflammatory cytokines [85]. This work confirmed that a peptide vaccine could effectively induce a protective immune response against *S. brasiliensis*.

*Sporothrix* glycoprotein Gp70 is a major adhesion factor on the fungal cell surface. The structure of Gp70 was screened by bioinformatic tools, and four peptides were identified and displayed on phage. Of the four, the phage displaying of the peptide KR (kpvqhalltplgldr) protected mice against infection with *S. globosa*, which is the most common species isolated in Northeast China [86]. Furthermore, the mice immunized with phage-KR produced high levels of IFN-γ + Th1 and IL-17 + Th17, indicating that the mechanism for protection by the recombinant phage may occur through the induction of a protective cell-mediated immune responses. However, serum from mice infected with *S. globosa* was also specifically recognized by phage-KR, which suggests that the humoral response may also be stimulated by the KR peptide [87]. Therefore, the phage-KR vaccine may function to enhance both protective cell-mediated and humoral immune responses, and this dual mechanism therapeutic represents a new and potentially safe strategy for the treatment of sporotrichosis.

### 3.5. Coccidioidomycosis

*Coccidioides* spp., are environmental pathogens that are responsible for the human respiratory disease Coccidioidomycosis, which occurs mainly in desert soils of the south-western United States and parts of Mexico and Central and South America [137,138,139]. Between 1998 and 2011, the number of reported cases of coccidioidomycosis increased from 5.3 to 42.6 per 100,000 inhabitants in the endemic region of the United States [140], which correlated with changes in weather and disturbances in soils. Recent studies also show that 17 to 29% of pneumonia cases in endemic regions are due to *Coccidioides* and the endemic regions are expanding [141,142]. Pursuing a vaccine for *Coccidioides* is not a new idea. In fact, a formalin killed spherule vaccine was developed and a clinical trial performed. From 1980–1985, 2876 patients were randomized to receive either three injections of 1.75 mg of FKS or placebo. Unfortunately, no differences in protection were observed [143,144], which may have been due to the low incidence of coccidioidomycosis during this period or the low dosing, which was required due to toxicity challenges [145].

Following the result with the whole cell vaccine, investigators turned their attentions to specific antigens for study and development. For example, in 2006, Pep1 was shown to be a cell wall dominant antigen that was protective in mice challenged with *C. posadasii*. By applying immunoproteomic and bioinformatic tools, investigators then identified five peptides from Pep1 that were predicted to have high affinity to MHC-II, and these peptides were able to induce IFN-γ by peptide-exposed lymphocytes [88]. Also in 2006, peptides from two additional proteins, Amn1 and Plb, from *C. pasadassi* were similarly demonstrated to stimulate IFN-γ production by T-cells [89]. In 2012, an epitope base vaccine (rEBV) [90] consisting of selected immunogenic peptides derived from Pep1, Amn1 and Plb was developed [88,89]. The rEBV significantly reduced fungal burdens, elevated IFN-γ and interleukin (IL)-17 production, as well as prolonged survival in vaccinated mice challenged with a lethal inoculum of *Coccidioides* compared to untreated infected mice [90].

More recently, a recombinant chimeric polypeptide vaccine (rCpa1) [91] was generated using the most immunogenic fragment of Ag2/Pra; the full lengths of Cs-Ag and Pmp1; and promiscuous, immunodominant T-cell epitopes derived from *Coccidioides posadasii*, Pep1, Amn1, and Plb. Administration of rCpa1 with the adjuvant GCP to both C57BL/6 and HLA- DR4 transgenic mice induced high levels of IL-17 in T-cell recall assays, earlier lung infiltration by activated Th1 and Th17, and increased the survival rates of mice lethally infected with *Coccidioides* compared to those that received GCPs alone [91].

Ag2/PRA has also been investigated for utilization in a dendritic cell vaccine for coccidioidomycosis [92,93,94,95,96]. In 2005, Ag2/PRA cDNA transfected into an immortalized dendritic JAWS II cell line was used to immunize mice challenged with *C. posadasii*, and the DC vaccine reduced the fungal burdens in both the lungs and spleens, and increased the amount of IFN-γ in the lung tissues of immunized compared to control mice [92]. Subsequently, Ag2/PRA-cDNA transfected bone marrow-derived dendritic cells administered intranasally were shown to migrate in blood, lung and thymus, and the vaccine induced Ag2/PRA-specific T cell response [94]. Safety testing revealed that intranasal immunization with an Ag2/PRA primary DC vaccine did not cause any illness or detectable injury to the mice. Immunization effectively induced the production of IFN, IL-4 and IL-17 in the lungs and lymph nodes of the vaccinated animals. Additionally, vaccination induced the production of all IgG isotypes [95]. These studies highlight the promising potential of this DC vaccine (Ag2-DC) for coccidioidomycosis as it effectively induced both cellular and humoral immune responses [92,94,95]

### 3.6. Pan-Fungal Vaccine

Although a vaccine to a single fungal pathogen would be heralded as a remarkable achievement, there is a desire to create a pan-fungal vaccine. The possibility for this is presented by work using monoclonal antibodies to conserved fungal cell surface epitopes to deliver cytocidal radiation [146,147,148]. However, peptide-based vaccines also hold great potential. In 2015, transgenic CD4^+^ T cells were used to identify an amino acid determinant within chaperone calnexin that was determined to be conserved across ascomycetes species [97]. The administration of calnexin in glucan particles elicited calnexin-specific CD4^+^ T cells, and vaccinated mice demonstrated resistance to infection by *Blastomyces dermatitidis, Histoplasma capsulatum, Pseudogymnoascus (Geomyces) destructans, Fonsecaea pedrosoi,* and *A. fumigatus* [97]. Similarly, the 13-mer peptide (LVVKNPAAHHAIS), which was generated from the conserved region amino acid determinant within chaperone calnexin, stimulated protective immune responses, and vaccination reduced the severity of infection with *B. dermatitidis* [97].

## 4. Chimeric Antigen Receptor (CAR) T-Cell Therapy

CAR T-cell therapy has primarily been used in combating diverse cancers [149,150], but there is a growing interest for its use in other diseases, including mycoses. CAR T-cell approaches use a patient’s T-cells to engineer them into chimeric cells that target both a specific antigen and activate other T cells. Currently, the target antigens are glycoproteins and lipids [151,152].

D-CAR is the second-generation of CAR T-cells therapy that targets Dectin-1, which is a C-type lectin receptor specific for β-glucan that is commonly expressed on the surface of diverse fungi [98]. These D-CAR T cells displayed specificity to Dectin-1 that was fused with CD28 and CD3-ζ such that effective T-cell activation signaling was generated. The administration of D-CAR T-cells to immunocompromised mice with invasive aspergillosis resulted in an increase in the levels of IFN-γ and impaired the growth of the *Aspergillus* [99]. Investigators in this area describe how CAR T-cell approaches can be dually impactful by designing the cells to target both the patient’s underlying cancer as well as a concomitant invasive fungal infection [100]. The use of engineered CAR T-cells engineering has been remarkably effective in cancer immunotherapy, and it has shown promise as a therapeutic for combating infections by viruses [153,154,155,156,157,158,159] and fungi [99], albeit it remains in early phase development for the treatment of mycoses.

## 5. Conclusions

Fungal diseases are widely neglected [160] and this extends to the development of vaccines to prevent and treat individuals with mycoses. Given that there are over 300 million people suffering from fungal infections annually with over 1.5 million of these dying [16,161,162], it is imperative that vaccine development be accelerated to combat these diseases. As one of the main factors that contribute to the increased frequency and severity of disease are defects cellular and/or humoral immunity [163], vaccine strategies must be safe and effective in hosts with intact and compromised immune systems. Advances in proteomics and system biology have facilitated the advancement of a number of vaccine proposals, particularly as they permit the localization of proteins and the characterization of their modifications, functions and interactions [164]. Predictions of epitope biology permit the rapid selection of peptides with expected immunogenicity that can be injected into hosts for presentation by professional APCs for subsequent recognition by B or T lymphocytes to induce a humoral or cellular immune response, respectively. The advancing studies with peptide vaccines and DC-peptide priming set the stage for future translation of these strategies from the bench to the bedside.

## Figures and Tables

**Table 1:** New vaccine proposals.

Fungi (Reference)	Vaccine (Peptide/Protein/Chimeric)	Immune Response	Results
*Paracoccidioides* [48,49,50,51,52,53,54,55,56,57,58]	Peptide vaccine (P10) P10 primary DC P10 primary monocyte derived-DC	CD4^+^ Th1 cell	Protection against i.t challenge, reduction of fungal burden both in immunosuppressed and immunocompetent mice, and efficacy of DNA vaccine; all tests were performed in animal models
	Prediction of sequence of epitopes from extracellular antigens	Potential to stimulate the immune response mediated by B cells and antibodies.	N.A.
*Aspergillus* [59,60]	Peptides from the protein Asp f1	Th1 cell	Peptides from Asp f1 stimulate production of Th1 cytokines.
*Candida* [33,61,62,63,64,65,66,67,68,69,70,71,72,73,74,75,76,77,78,79,80,81,82,83,84]	Fab and Met6 Peptides	Antibody	Mice immunized with either the Fba or Met6 peptide-DC vaccine had improved survival and reductions in fungal burdens in an immunosuppressed mouse model of disseminated candidiasis.
	14-mer Fab peptide conjugated each mimotopes from Met6 (PS2, PS31, PS28, PS55 and PS76) and	Specific antibody response	The peptides mimotopes induced a specific antibody response, and immunization with three of the peptide conjugate vaccines protected against disseminated candidiasis.
	18 peptides used to construct a multivalent recombinant protein	N.A.	N.A./requires specific HLA haplotypes to bind these particular peptide epitopes
	Recombinant protein (NDV-3 and NDV-3A)	B and T cells	Tested in Phase 1b/2a; one intramuscular dose was safe and NDV-3A was immunogenic and reduced frequency of recurrent vulvovaginal candidiasis (RVVC)
	Recombinant protein (NDV-3 and NDV-3A	Antibodies and CD4^+^ Th1 Cell	Vaccinated mice were protected against lethal *C. auris* infection.
*Sporothrix* [85,86,87]	Peptides (ZR1, ZR3, ZR3, ZR4, ZR5, ZR6, ZR7, ZR8)	CD4^+^ T cell	ZR3, ZR4 and ZR8 promoted cell proliferation in vitro. ZR8 induced IFN-γ, IL-17A and IL-1β, and showed protection against *S. brasiliensis* infection
	Phage displaying of the peptide KR	Th1 and Th17 cell and humoral immune response	Immunization with recombinant phage increased the survival rate of *S. globosa* infectedmice.
*Coccidioides* [88,89,90,91,92,93,94,95,96]	Peptides from the protein Pep1	-	Induced IFN-γ production when exposed to lymphocytes.
	Peptides from the proteins Amn1 and Plb	-	Induced IFN-γ production by T-cells
	recombinant T cell epitope-based vaccine (rEBV)	Th1, Th2, and Th17 cells	Mice immunized with rEBV had increased IFN-γ and IL-17 production, and they had significant reductions in fungal burden and prolongation of survival compared to nonvaccinated mice.
	Recombinant chimeric polypeptide vaccine (rCpa1)	Th1 and Th17 cell	rCap1 vaccination generated high levels of IL-17 in T-cell recall assays, earlier lung infiltration by activated Th1 and Th17, and increased the survival rates.
	Ag2/PRA-cDNA transfected DC	T cell	Vaccinated mice had lower fungal burdens and increased amounts of IFN-γ
	Ag2/PRA primary DC	T cell and IgG isotypes	Vaccinated mice did not show any illness or detectable injury and the immunization effectively induced IFN, IL-4 and IL-17 production
Pan fungal [97]	Calnexin peptide Recombinant calnexin (rCalnexin)	CD4^+^ Th1 and Th17 cells	rCalnexin formulated in GP reduced lung and spleen CFU in mice infected with *B. dermatitidis* or *Coccidioides posadasii* and prolonged survival. Calnexin peptide plus LPS delivery by i.v. route improved the expansion of calnexin-specific T cells.
Chimeric antigen receptor (CAR) T-cell therapy [98,99,100]	D-CAR T-cells	-	D-CAR^+^ T-cells controlled the *Aspergillus* infections in the presence of immunosuppressive drugs

LPS, Lipopolysaccharide; CFU, colony form unit; i.v, intravenous; rEBV, bacterium-expressed recombinant epitope-based vaccine; rCpa1, recombinant chimeric polypeptide vaccine; DC, dendritic cell; RVVC, recurrent vulvovaginal candidiasis; VVC, vulvovaginal candidiasis; GPs, yeast cell wall-derived glucan particles; N.A., not analyzed.
